# Self-reactive germline-like TCR alpha chains shared between blood and pancreas

**DOI:** 10.21203/rs.3.rs-3446917/v1

**Published:** 2023-10-20

**Authors:** Peter Linsley, Maki Nakayama, Elisa Balmas, Janice Chen, Fariba Pour, Shubham Bansal, Elisavet Serti, Cate Speake, Alberto Pugliese, Karen Cerosaletti

**Affiliations:** Benaroya Research Institute at Virginia Mason; Barbara Davis Center for Childhood Diabetes; Benaroya Research Institute; Benaroya Research Institute; Benaroya Research Institute; Benaroya Research Institute; The Henry Jackson Foundation; Benaroya Research Institute; City of Hope; Benaroya Research Institute, Seattle, WA

## Abstract

Human islet antigen reactive CD4 + memory T cells (IAR T cells) from peripheral blood have been studied extensively for their role in the pathogenesis of autoimmune type 1 diabetes (T1D). However, IAR T cells are rare, and it remains poorly understood how they affect T1D progression in the pancreas. Using single cell RNA-sequencing coupled with a multiplexed activation induced marker (AIM) enrichment assay, we identified paired TCR alpha/beta (*TRA/TRB*) T cell receptors (TCRs) in IAR T cells from the blood of healthy, at-risk, new onset, and established T1D donors. Using TCR sequences as barcodes, we measured infiltration of IAR T cells from blood into pancreas of organ donors with and without T1D. We detected extensive TCR sharing between IAR T cells from peripheral blood and pancreatic infiltrating T cells (PIT), with perfectly matched or single mismatched *TRA* junctions and *J gene* regions, comprising ~ 34% of unique IAR TCRs. PIT-matching IAR T cells had public *TRA* chains that showed increased use of germline-encoded residues in epitope engagement and a propensity for cross-reactivity. The link with T cells in the pancreas implicates autoreactive IAR T cells with shared *TRA* junctions and increased levels in blood with the prediabetic and new onset phases of T1D progression.

## Introduction

Many studies have investigated the role of islet antigen reactive (IAR) CD4 + and CD8 + T cells in peripheral blood of subjects with Type 1 Diabetes (T1D). Because of their specificity for islet autoantigens, IAR T cells are thought to be involved in autoimmune destruction of the pancreas. IAR T cells have been investigated for their role in disease mechanisms and as therapeutic targets and biomarkers for beta cell destruction ^1–7^. Levels of IAR T cells may be increased in the pancreas especially during the active phases of islet autoimmunity, which may last months to years before and after clinical diagnosis ^2,3^. Since pancreatic biopsy is not tenable in humans, most efforts have focused on peripheral blood. IAR CD4 + and CD8 + T cells are found in blood of at-risk and T1D subjects, but also often in healthy control (HC) subjects ^8–10^. Distinctive phenotypic properties of IAR T cells in T1D subjects suggest their association with disease, yet because IAR T cells are rare in the blood (1:30,000 to 1:160,000 for GAD65-specific IAR T cells) ^9^, it remains difficult to ascribe a role for them in pathogenesis in the pancreas.

A defining feature of T cells is the presence of T cell receptors (TCRs) on their cell surfaces. T cells proliferate in response to TCR recognition of antigenic peptides, resulting in clonal expansion of a population of cells with identical TCR sequences at both the nucleic acid and protein sequence levels and the same antigen specificity ^11^. Using paired TCR sequences identified by single cell RNA-sequencing (scRNA-seq), we previously demonstrated expanded clones of IAR T cells in blood of T1D patients ^12^. More recently, we identified a population of expanded IAR T cells with diverse specificities determined by TCRs with restricted *TRA* junctions and germline-constrained antigen recognition properties ^13^. In combination, these previous studies suggest the possibility that expanded IAR T cell TCR sequences in the blood of T1D patients represent the ontogeny of T cell autoimmune responses during disease. While this suggestion is attractive for both fundamental and translational investigations in T1D, evidence that IAR T cells with these characteristics are found in the pancreas is lacking. Studies have characterized islet infiltrating T cells in the pancreas from organ donors with T1D ^14–16^, but their linkage to IAR T cells in the blood is unclear.

In this study, we have examined IAR T cells in the pancreas by comparing TCR junction sequences between circulating IAR T cells and pancreatic infiltrating T cells (PIT). We show that a significant fraction of IAR TCRs in peripheral blood share matching *TRA* chains with PIT TCRs, and vice versa. We characterize PIT-matching TCRs and show that they have features of germline-like (innate) mode(s) of epitope engagement that are associated with TCRs displaying multi-specificity. Finally, we show expansion of PIT-matching *TRA* chains in blood prior to and near the time of T1D diagnosis, strengthening the link between IAR T cells in blood and pancreatic destruction.

## Results

### Isolation of TCR sequences from IAR and PIT T cells.

Our central hypothesis is that *in vivo* expansion of IAR T cells seen in peripheral blood ^12^ reflects autoimmune destruction of the pancreas during T1D. This hypothesis predicts the presence of IAR T cells in the pancreas during disease. To test this prediction, we utilized the extreme sequence diversity of TCRs to enable their use as “barcodes” for clonal populations of T cells recognizing specific antigens ^17^. We reasoned that because of the sequence diversity of TCRs and the rarity of IAR T cells in blood, it would be unlikely for individual IAR T cell TCR sequences to be present at high levels in PIT cells by chance. Significant overlap between IAR and PIT cell TCR sequences would therefore suggest a biological role for IAR T cells in the pancreas.

We utilized TCRs from IAR T cells isolated from the peripheral blood of two subject cohorts (**Table S1** and Materials and Methods). Cohort 1 was from our previous scRNA-seq comparisons of IAR T cells from HC subjects (n = 11), new onset T1D subjects (newT1D) < 100 days from diagnosis ( n = 26), and established T1D (T1D, n = 16 subjects) 12,13. Cohort 2 comprised additional HC (with no islet-directed autoantibodies) (n = 6) and newT1D subjects(n = 11); subjects with single autoantibodies (moderate risk for developing T1D, n = 8); and subjects having multiple autoantibodies (high risk, n = 6). We used Cohort 2 to validate and expand results obtained with Cohort 1.

We isolated IAR T cells from peripheral blood using an overnight activation induced marker (AIM) assay based on upregulation of CD154 and CD69 ^12,13^. For subjects in cohort 1, PBMC were stimulated with a pool of 35 immunodominant peptides from GAD65, IGRP, ZnT8, and preproinsulin restricted to the high-risk HLA class II DRB1*0401, *0301, or DQ8 molecules ^12,13^. PBMC from subjects in cohort 2 were stimulated in an HLA agnostic approach with overlapping peptide libraries (20 amino acids (AA) in length, 12 AA overlap) from the above islet proteins (**Table S1**). For both groups, CD154 + islet peptide activated cells were magnetically enriched, isolated as CD154 + CD69 + by single cell flow sorting and subjected to scRNA-seq to identify paired *TRA* and *TRB* chains in IAR T cells. In most experiments, we used TCRs from Cohort 1, comprising 4,331 TCR junctions (2,174 *TRA* and 2,136 *TRB*) from 2,784 cells and 53 donors (**Tables S1 and S2**). Where indicated, we combined Cohorts 1 and 2 and subset to donors with HLA-DRB*04 alleles, yielding 2,967 junctions (1,512 *TRA* and 1,433 *TRB*) from 1,873 cells and 43 subjects (**Table S1**).

For our initial experiments, we focused on Cohort 1. Approximately 90% of these subjects had high-risk DRB1*0401 HLA class II alleles, while ~ 10% had DRB1*0301 alleles. Using molecular cloning, lentiviral re-expression, and TCR functional assays, we identified multiple specific islet antigen epitopes that triggered 29/47 (~ 62%) of the TCRs tested from this cohort ^12,13^. These TCRs, therefore, represented IAR T cells from peripheral blood with a wide range of specificities.

For PIT cells, we utilized paired TCRs identified by Reverse Transcription PCR of islets or pancreatic tissues from organ donors provided by the Network for Pancreatic Organ Donors with Diabetes (nPOD) ^18^; a protocol at Vanderbilt University Medical Center/University of Pittsburgh; the Integrated Islet Distribution program (IIDP); and the Alberta Diabetes Institute Islet Core (ADI). PIT cell TCRs comprised a total of 9,798 unique *TRA* and *TRB* junctions from pancreatic tissues of autoantibody negative donors without diabetes, autoantibody positive donors representing prediabetic/preclinical stages of T1D, and T1D donors (1,785, 1,447 and 6,566, respectively). TCR junctions represented 4,706 T cells and 5,092 CD8 + T cells. PIT cell TCR sequences contained multiple perfectly matched amino acid junction sequences from preproinsulin reactive TCRs found in islets and peripheral blood ^19^. IAR T cell and PIT cell TCR sequences were subjected to several filtering steps before use in subsequent analyses (Materials and Methods) and are presented in **Table S2.** To maximize power, TCR sequences were not subset by subject group or cell type before analysis, unless otherwise indicated.

### TCRs from IAR T cells in blood share TRA chain sequence identity with TCRs from PIT cells.

**Figure 1:**
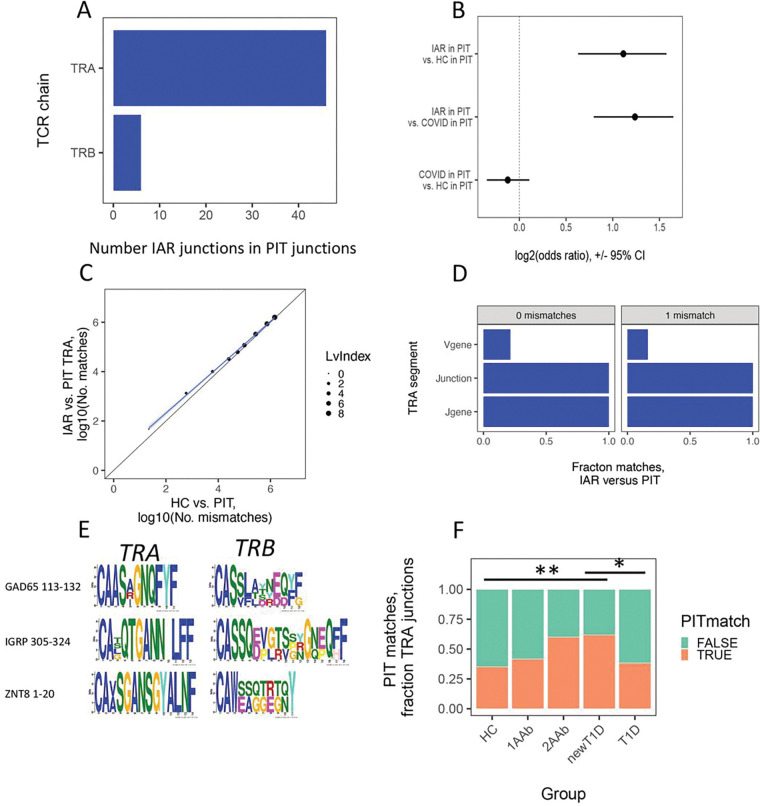
IAR T and PIT TCR sequence matching. A) Numbers of perfectly matched IAR T cell TCR *TRA* and *TRB* junctions from Cohort 1 in PIT TCRs. B) Numbers of PIT-matched versus non-matched *TRA* junctions from Cohort 1 IAR T cell TCRs compared with HC, and COVID-19 patient TCRs ^20^. Presented are log_2_ odds ratios calculated by Fischer’s exact test. Error bars, 95% confidence intervals. Dotted vertical line, log_2_ odds ratios of identity. C) Numbers of PIT matches in *TRA* junctions from Cohort 1 IAR T versus matches from a subset of HC donors ^20^, randomly size-matched to Cohort 1, over a range of mismatch values. Dot sizes, number of sequence pair matches with the indicated numbers of mismatches (Levenshtein index values). Diagonal line, equivalency line. Blue line, best fit line from linear modeling. Gray shading, 95% confidence intervals. D) PIT matches by different *TRA* junction segments in from Cohort 1 T cells (N = 1,389 *TRA* junctions). Lv0 and Lv1, Levenshtein index values: 0 (N = 47) and 1 (N = 1,342), respectively. E) MEME plots representing three groups of PIT-matching IAR sequences The topmost TCR in each group recognized ^13^ the indicated autoantigen epitopes: GAD65 113–132 (N = 4 TCRs); IGRP 305–324, (N=2); and ZNT8 1–20 (N = 2). F) Frequencies of PIT-matched and non-matched *TRA* junction numbers in IAR T cells by disease group. *TRA* chains from combined Cohort 1 and Cohort 2 were subset to HLA-DRB1*04 donors, and further separated by expanded and non-expanded cells (467 expanded and 1347 non-expanded cells). There were 12, 15, 145, 55 and 240 cells from 13 HC, 4 1AAb,3 2AAb, 11 newT1D, and 12 T1D donors, respectively. Differences were assessed using Fisher’s exact test. **, pAdj <1e-2; *, pAdj <5e-2. Comparisons not indicated did not differ significantly.

To test for TCR overlap, we first utilized string matches to test for perfectly matched recombined V-J or V-D-J junction sequence overlap between IAR and PIT cell TCRs. Unless otherwise noted, sequence comparisons were made at the amino acid level. We reasoned that perfectly matched junctions are most likely to reflect conserved function. This test showed multiple perfectly matched junction sequences, mainly *TRA* junctions compared to *TRB* junction matches (Fig. 1A). Because of the predominance of *TRA* chain matches, we focused subsequent analyses on *TRA* chains only. In initial experiments, we obtained essentially identical fractions of PIT-matching with IAR T in both CD4 + and CD8 + T cells. Since both cell types gave equivalent results, we pooled them to increase power in subsequent analyses. Overall, ~ 2.9% (47/1,606) of total IAR T cell *TRA* junctions perfectly matched PIT junctions; conversely, ~ 0.45% (44/9,798) of PIT cell TCRs matched IAR T cell TCRs. The distribution of perfect matches between different subject groups (HC, T1D and newT1D) and cell types (CD4+, CD8+) did not differ significantly from the distribution of total TCR populations from each group (p-value > 0.05, Fisher’s exact test). Thus, there were numerous perfectly matched *TRA* junction sequences shared between IAR T cells and PIT TCRs, even though the PIT TCRs were from different individuals than the IAR T cells.

### PIT-matched TRA junctions are enriched in IAR CD4 T cells relative to unselected TCR repertoires.

We hypothesized that junction sequence matching between IAR and PIT TCRs was greater than would occur by chance in an unselected repertoire. For comparison, we selected two PBMC repertoires from Su et al. (16 HC and 129 subjects with COVID-19, comprising 2,513 and 198,753 unique junctions for HC and COVID-19 patients, respectively) ^20^. To compare IAR PIT-matches with those in the unselected repertoires, we first down-sampled the unselected repertoires to match the size of unique IAR *TRA* junctions (N = 1,606). We then used Fisher’s exact test to compare numbers of perfectly PIT-matched to non-matched *TRA* junctions from IAR T cells versus unselected HC and COVID-19 T cells. We found more PIT matches with IAR *TRA* junctions than with junctions from unselected repertoires: there were 47 perfect matches with IAR junctions (2.9%) versus 22 (1.4%) for HC junctions. For IAR T cell junctions, this comparison yielded a log_2_ odds ratio > 1 (or > 2-fold in linear units), far outside the 95% confidence intervals (Fig. 1B) (p-value < 9e-6, Fisher’s exact test). In contrast, the log_2_ odds ratio was ~ 0 (or ~ 1 in linear units), and clearly within the 95% confidence intervals, for PIT-matched to non-matched junctions from COVID-19 patients versus HC subjects (Fig. 1B) (p-value > 0.05). Thus, there was a higher fraction of PIT TCR perfect matches with IAR T cells than unselected human TCR repertoires from HC and COVID-19 subjects.

TCRs from IAR T cells with mismatched junctions may also share functional properties. While we expect that shared function would be more likely with mismatches that conserve amino acid electrochemical properties, we have ignored these properties to simplify subsequent analyses. We tested for enrichment of IAR T cell *TRA* chains in PIT TCRs over a range of mismatch values by calculating pairwise Levenshtein index values. (The Levenshtein index is the number of residue changes needed to transform one sequence into another.) A plot of the numbers of PIT *TRA* junction matches with IAR versus HC T cells at different PIT mismatch thresholds (Fig. 1C) showed significant off-diagonal skewing in the direction of IAR TCRs (slope = 0.872, p-value (that the slope was not equal to 1) = 3e-3, by linear modeling). Thus, enrichment of PIT-matched to non-matched *TRA* junctions versus unselected HC TCRs was not limited to perfect sequence matches. Since 95% confidence intervals for PIT junction sequence overlaps with 0 and 1 mismatches showed greatest divergence between IAR T cell and HC TCRs, we chose to define IAR T cell *TRA* junctions with 0 and 1 mismatches as “PIT matches” in subsequent analyses.

### PIT-matched TRA junctions are paired with diverse TRB chains.

It was important to know whether PIT-matched *TRA* chains were paired with PIT-matched *TRB* chains. Although we observed a few *TRB* perfect matches (Fig. 1A), these were not paired with perfectly matched *TRA* chains. Likewise, perfectly PIT-matched *TRA* chains were paired with quite different *TRB* chain sequences (Table 1). We noted similar findings with single mismatched PIT junctions with TCRs of known specificity ^13^ (Table 1 and Fig. 1E). In this case, TCRs having specificity for one of several islet antigens and epitopes had *TRA* chains that more clearly matched PIT *TRA* chains than *TRB* chains. We conclude that PIT-matched *TRA* chains of TCRs with multiple antigen specificities were paired with diverse *TRB* chains. This diversity of *TRB* chains was not accounted for by different HLA specificities because most of the IAR TCRs used in this comparison shared MHC class II alleles.

### PIT TRA junction matches extend to include the J gene but not the V gene.

We hypothesized that different TCRs with greater sequence identity are more likely to share binding and functional specificity. To test the extent of sequence identity for IAR *TRA* chains with PIT matches, we extended the requirement for sequence matches into the V and *J gene* segments flanking matched junctions (Fig. 1D). Since the *V* and *J gene* segments in TCRs are much longer than junctions ^21^, they are less amenable to string match comparisons, prompting us to consider V and *J genes* as identical if they simply had the same name. For all junctions with 0 or 1 PIT mismatches, sequence identity extended towards the C terminus to include use of identical *J genes* (Fig. 1D). In contrast, sequence identity towards the N terminus was less marked, with only ~ 20–25% of matched junctions having identical *V genes*.

Since the TCR *V gene* contains the CDR1 and CDR2 regions that contact the MHC class I or class II molecules ^22^, it was possible that the low frequency of *V gene* identical matches reflected peptide presentation to PIT TCRs by different HLA molecules. IAR T cell TCRs were associated primarily with high-risk HLA genotypes ^13^, whereas PIT TCRs were from individuals having a wider variety of HLA genotypes. We therefore broke down the matching data from Fig. 1D by HLA class II DRB1 genotype. Individuals having 03:01, 04:01, 07:01 and pooled other DRB1 genotypes all showed similar frequencies of *V gene* identical matches (**Figure S1**). This led us to conclude that the low frequency of PIT matches in *V gene* regions was not attributable to peptide presentation by different HLA molecules.

We obtained results in Figs. 1A–1E from Cohort 1 (Materials and Methods). To validate these findings, we repeated these analyses with all samples in an independent cohort (Cohort 2). We obtained essentially identical results, demonstrating that our observations were not restricted to a single data set and therefore had a potentially broader range of islet specificities.

### PIT TRA matches with IAR T cell junctions are expanded early in disease.

We speculated that PIT *TRA* matches were related to T cell expansion and T1D progression. As a test, we quantified levels of PIT *TRA* matches in IAR TCR junctions found in > 1 cell (expanded) versus non-expanded *TRA* sequences in a cross-sectional analysis by disease group, initially using the Cohort 1 data set. To ensure that results were not biased by unequal numbers of junctions between disease groups, we randomly down sampled to equal numbers of junctions per group. This preliminary analysis revealed that the fraction of PIT *TRA* junction matches with expanded IAR T cells was significantly elevated, and the fraction of PIT *TRA* junction non-matches reduced, in newT1D subjects, relative to HC and T1D subjects.

To extend these results more broadly along the continuum of stages during the T1D disease process ^23^, we repeated the analysis using Combined cohorts (Fig. 1F). Because the peptide pools we used for Cohort 1 were biased towards HLA-DRB1*04 donors, we restricted our extended analysis for the combined data sets to this HLA type. The HLA-DRB1*04 subset of Combined cohorts comprised 1,873 cells and 2,967 junctions (1,512 *TRA* and 1,433 *TRB*) from 43 donors (**Tables S1 and S2**). Since we were able to sample relatively few 1AAb and 2AAb HLA-DRB1*04 subjects in Cohort 2, we performed this analysis without correction for different sample sizes. In cells with expanded TCRs, we saw a progressive increase in the fraction of PIT-matched junctions in IAR T cells collected from 1AAb, 2AAb and newT1D donors, then a decrease in T1D subjects to a similar level as in HC donors (Fig. 1F). There were no significant differences in PIT-matches between groups with non-expanded junctions.

To control for p-value inflation by increased numbers of junctions from relatively few donors, we repeated this analysis at the donor level (**Figure S2**). We observed the same trends as with junction frequency, though the effects were weaker and less significant. This suggests that these studies were underpowered at the donor (sample) level. Despite this limitation, these results supported the conclusion that an elevation of expanded PIT-matched IAR T cell *TRA* chains in blood occurred prior to the onset of clinical disease.

### PIT-matched TRA junctions were more germline-like and hydrophobic than non-matched chains.

We hypothesized that PIT-matched *TRA* junctions, like public TRA junctions ^13^, have different sequence and chemical properties than non-matched IAR T cell *TRA* junctions. As a first test, we asked whether PIT-matched *TRA* junctions were more likely to be shared between donors (public junctions) than non-matched junctions, as was expected by the different donor pools used to isolate IAR and PIT cells. Previously, we identified a population of IAR T cells with diverse specificities determined by TCRs with restricted TCR alpha junctions and germline-constrained antigen recognition properties ^13^. To determine how this population of public TCRs was related to PIT-matched/non-matched *TRA* junctions, we tested for junction sequence overlap. We found significant overlap between public IAR T cell and PIT-matched *TRA* junctions (p-value = 1.46e-14, hypergeometric distribution) but not private IAR T cell and PIT-matched *TRA* junctions (p-value > 0.05). Thus, there was strong overlap between cell populations with public and PIT-matching TCRs.

**Figure 2:**
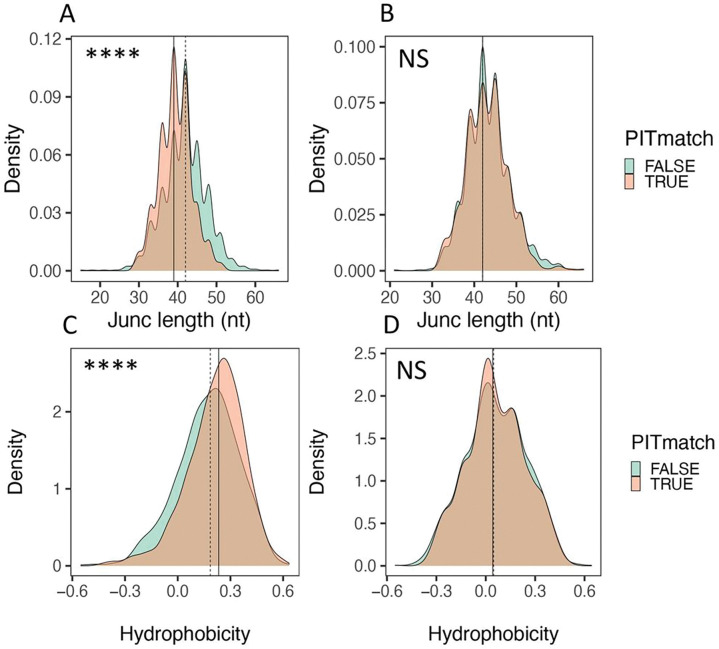
PIT-matched *TRA* junctions are shorter and more hydrophobic than PIT non-matched junctions. Distributions of *TRA* and *TRB* junction lengths (A and B) and hydrophobicity (C and D) calculated from amino acid sequences of *TRA* (A and C) and *TRB* junctions (B and D). The significance of differences was assessed using Kolmogorov-Smirnov tests. ****, pAdj <1e-4; **, pAdj <1e-2; NS, not significant. Solid vertical line, median value from PIT-matched junctions; dashed vertical line, median value from PIT-non-matched junctions.

As another test of differences between PIT-matched and non-matched junctions, we compared sequence features that have been correlated with TCR autoreactivity 13,24,25.(Fig. 2). We utilized density plots for these comparisons to elucidate the range of TCR junction lengths. We found that the distribution of *TRA* junctions was significantly left shifted (shorter) in PIT-matched junctions than in non-matched junctions (p-value < 1e-4, Kolmogorov–Smirnov test) (Fig. 2A). PIT-matched *TRA* junctions had a median length of ~ 39 nucleotides (nt), versus a median length of ~ 42 nt for PIT-non-matched junctions (i.e., 3 nt or 1 AA residue difference) (Fig. 2A). We did not see a significant difference with *TRB* junctions paired with PIT-matched and non-matched junctions; *TRB* junctions from both PIT-matched and non-matched *TRA* junctions had identical median length values of ~ 42 nt (Fig. 2B). Furthermore, *TRA* junctions (Fig. 2C), but not *TRB* junctions (Fig. 2D), were more hydrophobic in PIT-matched versus non-matched TCRs (p-value < 1e-4). PIT-matched and non-matched *TRA* junctions had median hydrophobicity values of 0.23 and 0.18, respectively, on the Eisenberg hydrophobicity scale ^26^, falling between values for proline and tyrosine (0.12 and 0.26, respectively). Thus, PIT-matched *TRA* junctions, but not their paired *TRB* junctions, were shorter, and more hydrophobic than PIT-non-matched *TRA* chains.

### Length differences between PIT-matched and non-matched TRA junctions map to peptide-contact regions.

We speculated that sequence features of PIT-matching *TRA* junctions were keys to the function(s) of IAR T cells having these TCRs. To further elucidate these sequence features, we used IMGT/HighV-QUEST ^27^ to characterize individual TCRs from their nucleotide (nt) sequences. We first determined the positions of single AA mismatches between PIT and IAR T cell *TRA* junctions. Since both *V* and *J gene* regions are germline-encoded, we predicted that PIT mismatches would not localize to these regions. Instead, we predicted that mismatches would instead occur primarily in the V-J recombination region, which is non-germline-encoded and varies in sequence between different TCRs. We found that mismatched residues were distributed symmetrically around amino acid position 4 (**Figure S3**). By comparison, the C terminal AA from *V genes* (3’ end) and the N terminal AA of *J genes* (5’ end), which mark the boundaries of germline-encoded residues in the respective gene segments, were centered at AA residues 3 and 5, respectively (**Figure S3**). Quantitatively, ~ 88% (1,178/1,342) of PIT-matched *TRA* junction mismatches were located between the 3’ends of *V genes* and the 5’ends of *J genes*. Normalizing the site of mismatches for junction length reduced variability but did not change the essential features of the results. Thus, PIT mismatches occurred primarily in the non-germline-encoded V-J recombination region.

We next focused our comparisons on regions important for TCR binding and function. Analogous to immunoglobulin molecules, TCR complementary determining regions (CDRs) convey the specificity for antigen and major histocompatibility complex molecules ^28^. A comparison of CDR1 region lengths identified using IMGT/HighV-QUEST 27 showed that the median CDR1 length was identical in PIT-matched and non-matched junctions, but the distribution was left-shifted significantly (i.e., CDR1 regions were shorter) for PIT-matched junctions (**Figure S4A**). CDR2 regions showed no length differences between PIT-matched and non-matched *TRA* chains (**Figure S4B**), whereas CDR3 regions, as expected since they comprise most of the junction sequences compared in Fig. 2, were shorter in in PIT-matched *TRA* chains (**Figure S4C**), by ~ 3 nt (1 AA). In other analyses, we found that framework (FR) regions FR1, FR2, FR3 and FR4 did not differ in length between PIT-matched and non-matched *TRA* chains, showing selectivity of the differences in CDR1 and CDR3 lengths.

We wished to determine the source(s) of junction length differences between PIT-matched and non-matched *TRA* chains. Since sequence variability of CDR3 regions mostly comes from random addition of nucleotides not encoded in the genome (N region), we compared N region lengths between the two groups of *TRA* chains. This comparison showed that PIT-matched junctions had median N region lengths of ~ 3 nt (1 AA) whereas PIT-non-matched junctions had median N region lengths of ~ 5 nt (1–2 AA) (**Figure S4D**). We also quantified contributions of the *V* gene and *J gene* segments adjacent to the N region. The 3’ end of the V region was slightly shorter in PIT-matched than non-matched *TRA* junctions (10 versus 11 nt) (**Figure S4E**) whereas the 5’ end of the J region was slightly longer (27 versus 25 nt) (**Figure S4F**). Thus, the difference in CDR3 lengths was a complex product of genome- and non-genome-encoded regions in PIT-matched junctions. Together, these analyses showed that PIT-matched and non-matched *TRA* junctions differed in *V gene* regions CDR1 and CDR3 that are important for peptide binding.

The difference in CDR1 region lengths was an unexpected result, since CDR1 regions are germline-encoded and not considered variable. We hypothesized that the basis for the shift in CDR1 length of PIT-matched *TRA* chains was enrichment for *TRA V gene* segments with shorter CDR1 regions. To test this, we compared enrichment of *V gene* segments in PIT-matched versus non-matched *TRA* chains (**Figure S5**). This showed that TRAV12–2*01 and TRAV41*01 *V genes* were significantly over-represented in PIT-matched IAR T cell *TRA* chains, whereas TRAV4*01 and TRAV26–2*01 were under-represented (or over-represented in PIT non-matched *TRA* chains). Over-represented *V genes*, TRAV12–2*01 and TRAV41*01, had CDR1 region lengths of 15 and 18 nt (5–6 AA). In contrast, under-represented *V genes*, TRAV4*01 and TRAV26–2*01, had 21 nt (7 AA) CDR1 regions. Thus, selective utilization of *V genes* with shorter CDR1 lengths was a likely explanation for enrichment for *TRA V gene* segments having shorter CDR1 regions in PIT-matched junctions.

### Determining predicted peptide binding contacts for PIT-matching and non-matching TRA chains.

CDR1 and CDR3 regions are involved in binding to peptides presented by MHC molecules ^22^. Based on this fact, we hypothesized that the observed length variation of these regions suggested altered peptide binding properties for PIT-matched *TRA* chains. As a test, we utilized molecular modeling to predict peptide binding residues in tri-molecular models of PIT-matched and PIT-non-matched TCRs complexed with peptide-class II MHC complexes. For modeling, we used TCRmodel2, which is based on the AI system, AlphaFold v2.3 ^29^, a newly described method that predicts protein structure with high accuracy at unmatched scale ^30^. TCRmodel2 uses focused databases of TCR and MHC sequences to expedite multiple sequence alignment feature building; optimization of the TCR template selection; and utilization of peptide–MHC complex structures as templates to improve modeling accuracy. For model input, we used paired *TRA* and *TRB* sequences from a set of 30 IAR TCRs with known specificity (16 PIT-matched and 14 PIT-non-matched TCRs), together with their cognate peptides ^13^; and sequences of HLA DRA*01:01 and DRB1*0401 MHC class II subunits ^31^.

**Figure 3:**
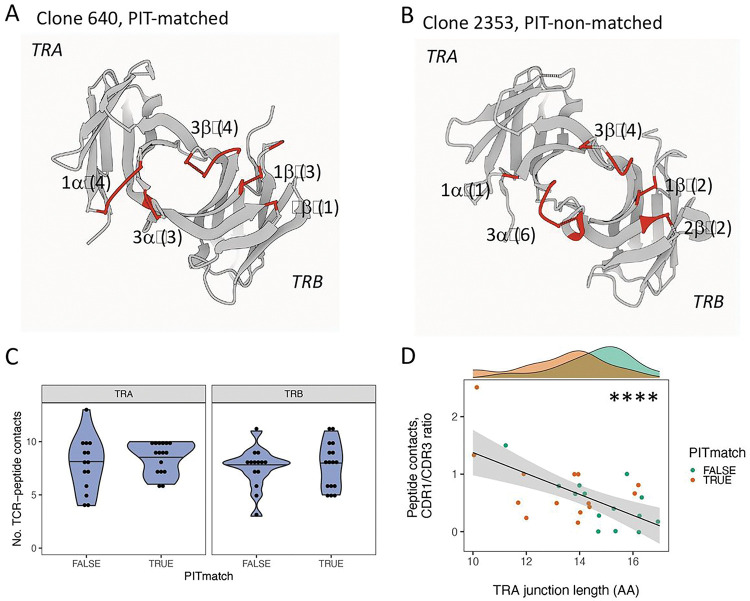
Increased ratios of germline-encoded to recombined peptide contacts in PIT-matched TCRs. A) Gaussian Surface representation of a model of Clone 640, a PIT-matching TCR with a 10 AA *TRA* junction (7 CDR contacts) paired with a 12 AA *TRB* junction (8 CDR contacts), that binds the GAD65 113–132 peptide ^56^ presented by the HLA DRB1*0401 class II molecule ^13^. The viewing plane is from the interface with the MHC molecules, which together with the peptide, have been removed from the representation for clarity. Labels indicate the CDR loops, with letters denoting the chain (α or β); and numbers, the CDR loop. Numbers in parentheses are the number of predicted peptide contacts with the CDR loop. B) A model of Clone 2353, a PIT-non-matching TCR with a 17AA *TRA* junction (7 CDR contacts) paired with a 14 AA *TRB* junction (8 CDR contacts), that binds the IGRP305–324 peptide presented by the HLA DRB1*0401 class II molecule. C) The number of total peptide contacts for either the *TRA* or *TRB* chains did not differ significantly between PIT-matched and PIT-non-matched TCRs (p-value >0.05, Wilcox test). D) Ratios of *TRA* CDR1 to CDR3 peptide contacts decreased significantly with increasing *TRA* junction length. ****, p-value < 1e-4 for a slope of 0, calculated using linear modeling.

From best fit models for each TCR, we identified likely peptide contact residues (TCR residues < 5 Å in distance from the bound peptide chain) and mapped these to *TRA* and *TRB* sequence features. We first visually compared TCR-peptide contact residues in representations of PIT-matched and PIT-non-matched TCRs with extremes in *TRA* junction length (Fig. 3A–B). This comparison showed that Clone 604 (10 AA, PIT-matched) had more predicted peptide contacts that mapped to CDR1, and fewer that mapped to CDR3, than Clone 2253 (17 AA, PIT-non-matched). To extend these observations to the larger data set, we compared the numbers of TCR contacts of each chain from the set of 30 IAR TCRs (**Table S3**). We displayed the numbers of contacts in different sequence features in both chains from each TCR as a function of *TRA* and *TRB* junction lengths (**Figure S6**). As expected, the *TRA* and *TRB* CDR3 regions contributed the most peptide contacts. There was a significant positive relationship between the number of peptide contacts mapping to the *TRA* CDR3 region and *TRA* junction length, and a weaker negative relationship with the *TRB* CDR3 region (**Figure S6**). While the number of contacts was low, there was also a negative relationship between the *TRA* CDR1 region and *TRA* junction length.

### Increased dependence on germline-encoded residues for binding of shorter and PIT-matching TCRs.

The variation in contact residues by junction length (and PIT match) could indicate either differences in overall number of contacts, or different ratios of contacts in different regions. To test these possibilities, we first compared the overall number of TCR contacts by TCR chain in PIT-matched versus PIT-non-matched TCRs. This comparison showed no significant difference in overall peptide contacts in either *TRA* or *TRB* chains between the two groups (Fig. 3C). In contrast, the ratio of *TRA* CDR1 to CDR3 contacts varied significantly with *TRA* junction length (p-value < 1e-4 for line slope, by linear modeling), while maintaining the separation in length between PIT-matched and PIT-non-matched junctions (Fig. 3D). Thus, peptide recognition by PIT-matched TCRs shows a trend towards greater reliance on the germline-encoded residues in the *TRA* CDR1 region than do PIT-non-matched TCRs, which in turn show greater reliance on CDR3 residues generated by V(D)J recombination.

Concomitant with the increase in the ratio of CDR1 to CDR3 contacts, PIT-matched *TRA* chains also showed a decrease in CDR1 region length (**Figure S4A**). This incongruity could suggest a change in physical properties of the shorter CDR1 regions to make them more amenable to close peptide contacts. Thus, it may be important that PIT-matched CDR1 regions were weakly more hydrophobic than PIT-non-matched regions (p-value = 0.049, one-sided Kolmogorov–Smirnov test).

### TCRs with PIT-matching TRA junctions show evidence of multi-specificity.

**Figure 4:**
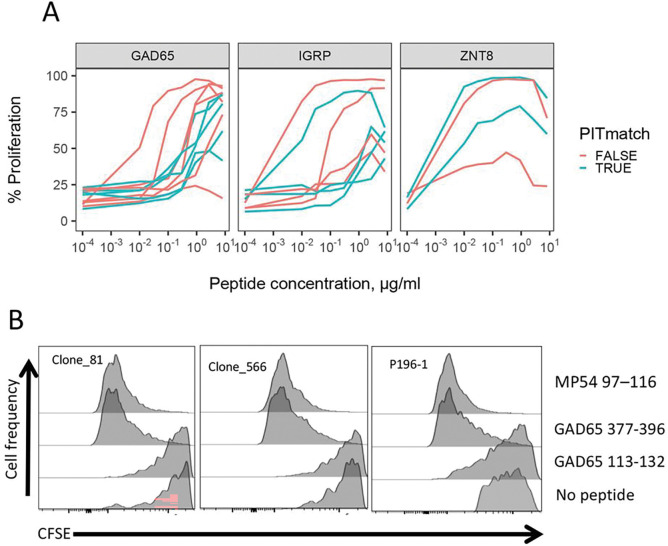
Functional properties of selected PIT-matching and non-matching *TRA* chains of known specificity. A) Dose response curves for TCRs recognizing GAD65, IGRP and ZNT8 epitopes ^13^. Curves for multiple epitopes per target antigen were pooled to increase power; examination of curves for individual epitopes yielded conclusions consistent with the pooled data. B) Cross-reactivity of TCRs with PIT-matching *TRA* junctions (Table 3) for autoimmune (GAD65) and viral (influenza M protein) epitopes in a CFSE proliferation assay.

We hypothesized that different peptide binding modes of PIT-matched and non-matched *TRA* junctions would lead to altered peptide recognition properties, such as strength and/or specificity of binding. As a measure of binding strength, we compared the functional avidities of TCRs having PIT-matched and non-matched *TRA* junctions specific for different peptide epitopes as described previously ^13^. We plotted cell proliferation, as measured by CFSE ^13^, versus peptide concentration (Fig. 4A) for the subset of TCRs having PIT-matching or non-matching *TRA* junctions (Table 2). To increase power, we aggregated results with different peptides from each of three different islet antigens (GAD, IGRP and ZNT8). These results showed wide variability in range of dose responses, but no consistent differences between TCRs with PIT-matched and non-matched *TRA* junctions. EC50 values ^13^ for aggregated GAD65- and IGRP-specific TCRs with PIT-matched and non-matched *TRA* junctions did not differ significantly by unpaired Wilcox tests; since there were only two ZNT8 TCRs, a p-value could not be calculated. We obtained similar overall results when individual peptides were considered. These data do not support there being large differences in functional avidity between TCRs with PIT-matched and non-matched *TRA* junctions.

Based on these results, we next hypothesized that TCRs with PIT-matched and non-matched *TRA* junctions differed in peptide binding specificity. This was supported by our previous finding of multi-specificity of some public IAR TCRs 13. Though limited in number (n = 3), the frequency of these multi-specific clones was higher in public compared with private clones ^13^. In the present studies, we noted that *TRA* chains from all three of these multi-specific TCRs were PIT-matching. Indeed, multi-specific *TRA* junctions were modestly more frequent in PIT-matching than non-matching *TRA* junctions (one-sided p-value = 0.030, Fisher’s exact test).

Two of these PIT-matching multi-specific TCRs (Clones 81 and 566) recognized non-overlapping GAD65 epitopes ^13^. In parallel and independent experiments, we unexpectedly found that the *TRA* chain of a TCR (P196–1) from Influenza A/MP54- reactive CD4 + T cells perfectly matched the *TRA* chain from Clones 81 and 566 (Table 3). Furthermore, P196–1 had *TRB* chains that differed from Clones 81 and 566 at only a single AA position (Table 3). This sequence similarity led us to reason that closely related Clone 81, Clone 566 and P196–1 were all multi-specific. As a test, we compared the ability of activating peptides (MP54 97–116 and GAD65 377–396), a non-activating peptide (GAD65 113–132) to trigger proliferation of TCR-transduced primary CD4 + T cells (Fig. 4B). We found that both MP54 97–116 and GAD65 377–396 peptides identically activated Clone 81, Clone 566, and P196–1 TCRs. Thus, these TCRs with PIT-matched *TRA* junctions were all multi-specific, supporting our hypothesis that TCR sequence features, including shorter and more hydrophobic *TRA* junctions, are linked to an inherent tendency for multi-specificity.

To elucidate possible mechanisms of cross-reactivity of the Clone 81 TCR for the MP54 97–116 and GAD65 377–396 peptides, we constructed molecular models ^32^ of the Clone 81 TCR ^13^ and complexes of these peptides together with HLA-DRA1*0101/DRB1*0401 molecules ^31^. The cognate GAD65 377–396 (**HKWKLSGVERAN**SVTWNPHK, where bold font denotes core sequence complexed with the TCR) and MP74 97–116 (VKLYR**KLKREITFHGA**KEIS) peptides did not show compelling sequence similarity by multiple sequence alignment. They also showed no evidence of structure ^33^ or aromatic side chain ^34^ conservation characterizing “hotspots” of molecular mimicry used by some crossreactive TCRs. Despite their low degree of peptide sequence similarity, molecular models of Clone 81 TCR with both peptides yielded identical scores of 0.88. In addition, *TRA* and *TRB* chains in both models showed similar predicted topography in their interactions with peptide-MHC class II complexes (**Figure S7A-B**). Overlapping but non-identical residues in the modeled TCR *TRA* and *TRB* chain CDR1, CDR2 and CDR3 regions contacted the different peptides (**Figure S7C**). Alignment of predicted structures of the *TRA* (**Figure S7D**) and *TRB* (**Figure S7E**) chains from models made with the different peptides showed nearly perfect superposition except in the CDR3 regions. This suggests that the PIT-matched Clone 81 TCR accommodates quite different peptide sequences through interactions involving conformationally conserved CDR1 (and CDR2 chains for *TRB*) regions, together with more variable CDR3 regions.

### Multi-specific TCRs shared sequence features with PIT-matched TCRs.

**Figure 5:**
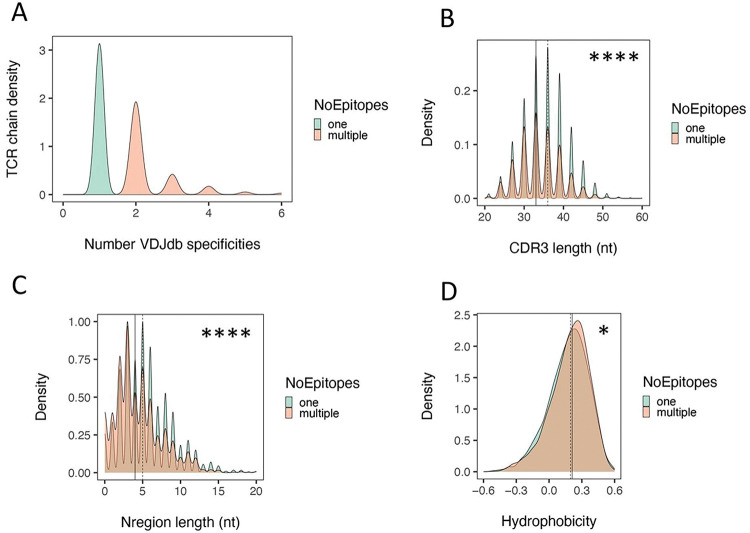
*TRA* junctions from cross-reactive TCRs in the *VDJdb* database shared junction sequence features with PIT-matched *TRA* junctions. A) Density of *TRA* chains from TCRs with one versus multiple specificities in *VDJdb*. There were N = 17,826 unique *TRA* chains from TCRs with single specificity and N = 1,664 with multiple specificities. B) Distribution of TRA CDR3 nt lengths from TCRs with one versus multiple specificities; C) Distribution of *TRA* N region nt lengths. D) Distribution of *TRA* junction AA hydrophobicity. Solid vertical lines, median values from *TRA* chains from multi-specific TCRs; dashed vertical lines, median values from TCRs with single reported specificities. Significance of differences in distributions were assessed using Kolmogorov-Smirnov tests. ****, pAdj <1e-4; *; pAdj <0.05.

We wished to test in grerater depth our hypothesis that PIT-matched and non-matched *TRA* junctions differed in peptide binding specificity. Recognizing that the number of known multi-specific TCRs from IAR T cells was too small at present to enable firm population-based conclusions about multi-specificity, we took an alternative approach to test our hypothesis. In other experiments, we observed that *VDJdb*, a curated public database of TCRs with known specificities ^35^, contains TCR sequences that recognize both single and multiple peptide epitopes. While we recognized that these designations were likely biased, with some TCRs being designated as single specificity because they were understudied, and others being designated as multi-specific on the basis of reactivity with closely related peptides. We reasoned, however that these errors would tend to offset each other, and that the size and scope of this database would provide a more comprehensive source of specific and multi-specific TCRs, which could be used to test their junction sequence features. There were N = 17,826 unique *TRA* chains in *VDJdb* that recognized single epitopes and N = 1,669 *TRA* chains that recognized multiple epitopes (Fig. 5A). Since nt sequences used to identify junction sequence features are not readily available for *VDJdb* TCRs, we utilized the software package, *Stitchr*, to produce complete TCR cDNAs from V/J/CDR3 AA sequences ^36^. We then used IMGT/HighV-QUEST ^27^ to identify TCR sequence features from the predicted cDNA sequences. We found that *TRA* CDR3 regions (Fig. 5B) from TCRs that recognized multiple epitopes were significantly shorter (by ~ 3 nt in median length, or ~ 1 AA) than junctions from TCRs that recognized single epitopes. We found equivalent differences in junction AA sequences, indicating that the results were independent of *Stitchr*. Likewise, TRA chains from multi-specific TCRs contained fewer non-templated (N region) nucleotides (~ 1 nt in median length) than TCRs with single specificity (Fig. 5C). Finally, *TRA* junction amino acid sequences from multi-specific TCRs were more hydrophobic in TCRs that recognized multiple epitopes (Fig. 5D). None of these differences were seen with *TRB* chains (**Figure S8 A-D**). Thus, multi-specific TCRs from *VDJdb* shared multiple sequence features with PIT-matched TCRs, including shorter *TRA* but not *TRB* chains, that had fewer N region nt, and encoded more hydrophobic AA sequences.

## Discussion

It has long been unclear whether and how rare autoreactive cells in peripheral blood represent autoimmunity in in target organs. In other words, are these cells drivers or passengers in autoimmune processes? To help resolve this question, we show here that a significant fraction of IAR TCRs from peripheral blood share matching *TRA* chains with PIT TCRs, and vice versa. Thus, a subset of IAR T cells shares a TCR chain with TCRs present in the pancreas. Since there are no familial or genetic relationships between the Cohort 1 and Cohort 2 blood donors and the nPOD organ donors, the TCR chain sharing we demonstrate involves public sequences. We also show that frequencies of PIT-matching *TRA* chains in blood increase prior to the time of diagnosis, suggesting a temporal linkage of levels of PIT-matching *TRA* chains in blood with disease progression. Shared *TRB* chains between peripheral blood and insulin-reactive TCRs from the pancreas were reported recently ^19^. These combined studies place autoreactive TCR chains at the scene of disease (the pancreas) at the right time (early in the disease process) to influence disease development.

However, we must emphasize that, at present, we do not know how many of these autoreactive TCR chains are associated with functional autoreactivity versus β-cells. At present, there is no way to predict with certainty that TCRs share specificity unless they show complete identity of both chains. In the present case, PIT-matching *TRA* chains shared quite divergent *TRB* chains, and it remains unknown what fraction of these TCRs recognizes autoantigens. Nonetheless, our demonstration of *TRA* chain sharing strengthens the link between IAR T cells in blood and autoimmune destruction of the pancreas.

T cell specificity is key to cellular immunity ^37^. Paradoxically, TCR cross-reactivity is necessary because the diversity of TCRs, while huge, is dwarfed by the vast array of potential foreign peptide-MHC complexes ^38,39^. Cross-reactivity expands the potential range of a given repertoire towards foreign antigens, but it comes at the expense of greater potential for increased self-reactivity or autoimmunity. These considerations emphasize the importance of understanding of sequence and structural features determining TCR cross-reactivity. Unfortunately, at present such an understanding remains incomplete ^37,40^. Our data show that cross-reactive (multi-specific) TCRs share sequence features with PIT-matched TCRs, namely shorter and more hydrophobic *TRA* junctions and diverse *TRB* chains, reminiscent of a previous report in mouse ^41^. Supporting this conclusion, the few islet-specific TCRs for which we have demonstrated cross-reactivity are PIT-matched TCRs. Earlier work demonstrated shortened ^25^ and more hydrophobic ^24^ *TRB* chains in bulk T cell subsets from T1D subjects, and suggested that these features are important in development of self-reactive T cells.

TCR CDR3 regions form most of the primary contacts with peptide epitopes presented by MHC molecules ^22^. For most TCRs, *TRB* CDR3 contacts are most numerous ^42^, although there are multiple examples of MHC class I-restricted TCRs that predominantly utilize germline-like *TRA* CDR3 regions ^43–47^. Less is known about such *TRA*-centric, MHC class II-restricted TCRs, such as those we have observed. One of the *V genes* enriched in PIT-matched TRA chains (**Figure S5**), TRAV12–2, is also a feature of multiple MHC class I-restricted TCRs ^43,44,46,47^. The other *V gene* enriched in PIT-matched *TRA* chains was TRAV21–2, which is associated with celiac disease, while paired with TRBV9 chains Our findings suggest that utilization of *TRA*-centric binding is a previously undescribed but widespread feature of IAR TCRs, and perhaps MHC class II-restricted TCRs in general.

Molecular modeling showed that germline-encoded CDR1, rather than CDR3, residues from TRAV12–2 contributed critically to binding of an immunodominant TCR to Yellow fever virus ^46^. This feature may contribute to the high precursor frequency and immunodominance of this TCR ^46^. PIT-matching IAR T cell *TRA* chains also employed less non-templated and more germline-like (innate) mode(s) of epitope engagement. *TRA* CDR3 regions of PIT-matching IAR varied from the genome by relatively few N region nucleotide sequences. In addition, PIT-matching IAR T cell *TRA* chains showed shorter CDR3 regions and longer *TRAJ* segments than non-matching IAR T cell *TRA* chains. TCR CDR1 regions contact both peptide epitope and MHC residues, while CDR2 regions generally contact only MHC residues ^22^. Thus, TCR regions important for peptide binding were shortened in PIT-matching *TRA* chains and had altered peptide binding properties. These PIT-matching *TRA* chains were also more hydrophobic at the amino acid level, suggesting partner agnostic interactioins via the hydrophobic effect versus more residue specific effects such as hydrogen bonding. Our results also suggest that these shorter and more hydrophobic, germline-like, *TRA* chains are a feature of many cross-reactive TCRs. While some TCRs that predominantly utilizing germline-like *TRA* CDR3 regions are cross-reactive ^49^, this has not been previously demonstrated on the global level we show here.

One caveat to our study is that we have addressed only sharing of public TCRs. This was by necessity, since TCR sequences from IAR and PIT T cells from the same individuals were not available. While this feature enhances broadness of potential translational applications, it also limits broad mechanistic conclusions. Future studies may be able to address this weakness by testing of additional nPOD tissues ^18^, including spleen and pancreas draining lymph nodes, from the same donors used for PIT TCR identification. Another weakness in our studies is that it is underpowered with respect to numbers of subjects studied. This is apparent in our examination of individual donor frequencies between PIT-matched and non-matched *TRA* junctions (compare Fig. 1F versus **Figure S2**). Our study is also underpowered for autoreactive TCRs of known specificity needed to directly test our hypothesis that PIT-matched TCRs tend to be multi-specific. Such a test is not possible at present because the numbers of IAR T cell TCRs with demonstrated specificity remains small. While we used *VDJdb* sequences of different specificities as an indirect test, a. more comprehensive collection of autoreactive TCRs with known specificity will be needed to further clarify the situation.

Our studies have potential translational implications. Typically, studies on TCRs as biomarkers have taken a reductionist approach by focusing on one or a few TCRs to monitor disease progression and/or response to therapy 19,50,51. In contrast to this approach, our broader-based studies place many TCRs with different islet antigen specificities and distinct *TRA* sequence features in both the pancreas and blood in early stages of T1D progression. Together, these new findings suggest that an alternative to the reductionist approach for translational studies is to consider autoreactivity as a web of T cell specificities and potential cross reactivities. Our findings provide a basis for expansion of future biomarker studies to include a broader network of autoreactive and potentially multi-specific TCRs.

## Materials and Methods

### Repository information.

Code and data for generating Figures, as well as pdb files of molecular models, are available at https://github.com/BenaroyaResearch/. Profiles yielding TCRs were deposited in the GEO repository (https://www.ncbi.nlm.nih.gov/geo/) under accession numbers GSE182870 (Cohort 1) and GSEXXXXX (Cohort 2).

### Study approval.

Protocols for these studies were approved by the Institutional Review Board of Benaroya Research Institute (IRB7109–332). Protocols for the clinical studies were approved under the auspices of NCT00129259 for the AbATE trial ^52^ and NCT00515099 for the START study.^53^

### Experimental methods

Methods are available as Supplemental Methods.

## Figures and Tables

**Table 1:** TCRs sharing perfectly matched *TRA* chains between IAR and PIT T cells. ^A^

	*TRA* chain			*TRB* chain		
Cells	*V gene*	Junction	*J gene*	*V gene*	Junction	*J gene*
IAR	TRAV1-2	CAVRMNTGFQKLVF	TRAJ8	TRBV11-2	CASSFGGGATDTQYF	TRBJ2-3
PIT	TRAV1-2	CAVRMNTGFQKLVF	TRAJ8	**TRBV9** ^B^	CASS**V**G**MDPGLGYNEQF**F	**TRBJ2-1**
IAR	TRAV12-1	CVVNDQAGTALIF	TRAJ15	TRBV7-2	CASSLDAGRNSPLHF	TRBJ1-6
PIT	TRAV12-1	CVVNDQAGTALIF	TRAJ15	**TRBV20-1**	C**SARGYNSYEQY**F	**TRBJ2-7**
IAR	TRAV12-1	CVVQGGSYIPTF	TRAJ6	TRBV5-4	CASSLVTSGENEQFF	TRBJ2-1
PIT	TRAV12-1	CVVQGGSYIPTF	TRAJ6	ND ^C^		
IAR	TRAV12-2	CAVNQAGTALIF	TRAJ15	TRBV28	CASSFGSGADYGYTF	TRBJ1-2
PIT	TRAV12-2	CAVNQAGTALIF	TRAJ15	**TRBV29-1**	C**SVFDWDRGPGELF**F	**TRBJ2-2**
IAR	TRAV12-2	CAVRSNFGNEKLTF	TRAJ48	TRBV19	CASGTDSY-EQYF	TRBJ2-7
PIT	TRAV12-2	CAVRSNFGNEKLTF	TRAJ48	**TRBV28**	CAS**R**T**TGGT**E**AF**F	**TRBJ1-1**
IAR	TRAV13-1	CAASIGTGTASKLTF	TRAJ44	TRBV9	CASSVA-GGGY-EQYF	TRBJ2-7
PIT	TRAV13-1	CAASIGTGTASKLTF	TRAJ44	**TRBV24-1**	CA**T**S**DPS**GGGG**N**EQ**F**F	**TRBJ2-1**
IAR	TRAV41	CAASNTGNQFYF	TRAJ49	TRBV28	CAIGGRVYNEQFF	TRBJ2-1
PIT	TRAV41	CAASNTGNQFYF	TRAJ49	**TRBV5-1**	CA**SS**G**SN**Y**GYT**-F	**TRBJ1-2**
IAR	TRAV5	CAERGLTGGGNKLTF	TRAJ10	TRBV9	CASSVGGDFYNEQFF	TRBJ2-1
PIT	TRAV5	CAERGLTGGGNKLTF	TRAJ10	**TRBV12-5**	CAS**GLTRGSTDT**Q**Y**F	**TRBJ2-3**
IAR	TRAV8-2	CVVSGGSNYKLTF	TRAJ53	TRBV29-1	CSAHGGGGT-EAFF	TRBJ1-1
PIT	TRAV8-2	CVVSGGSNYKLTF	TRAJ53	**TRBV6-1**	CAS**SQ**G**TPQYN**E**Q**FF	**TRBJ2-1**
IAR	TRAV8-3	CAVGPTGTASKLTF	TRAJ44	TRBV7-6	CASSTNHQ------ETQYF	TRBJ2-5
PIT	TRAV8-3	CAVGPTGTASKLTF	TRAJ44	**TRBV3-1**	CASS**GTGTGGLSPQ**ETQYF	**TRBJ2-5**

A Shown are amino acid sequence comparisons of unique IAR and PIT T cell TCRs. Dashes indicate gaps.

B Bold font indicates a mismatch with the IAR reference sequence (top row of each pair).

C ND, not determined

**Table 2:** TCRs sharing single mismatched *TRA* chains between PIT and IAR T cells of known specificity. ^A^

	TRA chain			TRB chain		
specificity	*V gene*	Junction	*J gene*	*V gene*	Junction	J gene
GADp15	TRAV41	CAAA-GNQFYF	TRAJ49	TRBV12-4	CASSFT-YNEQFF	TRBJ2-1
	**TRAV29/DV5** ^B^	CAA**R**-GNQFYF	TRAJ49	**TRBV6-2**	CASS**LLNLD**NEQFF	TRBJ2-1
	**TRAV21**	CAA**I**-GNQFYF	TRAJ49	**TRBV29-1**	C**SVLRDRASY**EQYF	**TRBJ2-7**
	**TRAV29/DV5**	CAA**SA**GNQFYF	TRAJ49	**TRBV4-1**	CASS**LAATRDDYGYT**F	**TRBJ1-2**
IGRP39	TRAV25	CAGQTGANNLFF	TRAJ36	TRBV4-3	CASSQEVGTVPNQPQHF-	TRBJ1-5
	**TRAV16**	CA**L**QTGANNLFF	TRAJ36			
	**TRAV13-1**	CA**T**QTGANNLFF	TRAJ36	TRBV9	CASS**VGR-SSYNEQF**F	TRBJ2-1
	**TRAV24**	CA**S**QTGANNLFF	TRAJ36	**TRBV4-1**	CASS**QDPLTSGRGNEQF**F	TRBJ2-1
ZNP1	TRAV13-1	CAASGANSGYALNF	TRAJ41	TRBV30	CAWSAQGETQYF	TRBJ2-5
	**TRAV8-4**	CA**V**SGANSGYALNF	TRAJ41	TRBV30	CAW**ESGTRGNYGYT**F	**TRBJ1-2**

A Shown are amino acid sequence comparisons of TCRs having single mismatched *TRA* junctions between IAR and PIT TCRs. IAR TCRs of the indicated specificities were used as reference sequences.

B Bold font indicates a mismatch with the IAR reference sequence (top row of each pair).

**Table 3:** Mismatched TRB chains from multi-specific TCRs with perfectly matched TRA chains. ^A^

Clone ID	Specificity	Epitope	*TRBV*-gene	Junction	*TRBJ*-gene
Clone_81	Islet	GAD65 377-396	TRBV12-4	CASS**P**QGGNTEAFF	TRBJ1-1
Clone_566	Islet	GAD65 377-396	TRBV12-4	CASS**V**QGGNTEAFF	TRBJ1-1
P196-1	Influenza	MP54 97-116	TRBV12-4	CASS**L**QGGNTEAFF	TRBJ1-1

A Shown are mismatched *TRB* chain amino acid sequence comparisons of multi-specific TCRs that recognized both GAD65 and MP54 peptides. All TCRs had identical *TRA* chains (TRAV29DV5-CAASRYSGGGADGLTF-TRAJ45). Sequences of GAD65 377-396 ^54^ (H**KWKLSGVERAN**SVTWNPHK, where bold font denotes core sequence complexed with the TCR) and MP74 97-116 ^55^ (VKLYR**KLKREITFHGA**KEIS) peptides showed minimal sequence similarity by multiple sequence alignment.

B Bold font indicates mismatches between the three *TRB* chains.
